# Modelling the impact of injection time on the bolus shapes in PET-MRI AIF Conversion

**DOI:** 10.1186/2197-7364-1-S1-A54

**Published:** 2014-07-29

**Authors:** Hasan Sari, Kjell Erlandsson, Anna Barnes, David Atkinson, Simon Arridge, Sebastien Ourselin, Brian Hutton

**Affiliations:** Institute of Nuclear Medicine, University College London / University College London Hospitals, London, NW1 2BU UK; Center for Medical Imaging, University College London, London, NW1 2PG UK; Center for Medical Image Computing, University College London, London, WC1E 6BT UK

With the introduction of combined PET/MRI systems, AIF conversion can be made under certain circumstances (see [1]). We propose a model that allows modification of the injection parameters in the AIF fit to account for differences caused by different injection durations [2].

Brain ^18^F-Choline PET and DSC-MRI data were obtained using Siemens mMR. The MR contrast agent was injected with a rate of 4ml/sec and the PET tracer was injected manually. Perfusion Mismatch Analyzer [3] was used to extract the MRI-AIF. Carotid arteries were segmented on a post contrast MPRAGE image. PET frames were registered onto this MPRAGE image using rigid registration and partial volume correction was done using the iterative Yang method [4]. The AIFs were fitted using a convolution of a ‘double Butterworth’ function, representing the injection, with a tri-exponential function representing the elimination [Eq. 1]. The bolus shape can be adjusted by changing Δτ (τ_2_ - τ_1_). This was tested with a population based MRI AIF [5], as well as with clinical data.1

where

For the population based input function, Figure 1 shows that when Δτ was increased, lower and wider peaks were seen, and with decreased Δτ, higher but narrower peaks were observed. Figure 2 shows that the function fits both clinical PET and MRI AIFs well. Values of τ_1_ and τ_2_ were changed to modify the MRI-AIF and Figure 3 shows the modified MRI-AIF together with the original fitted PET-AIF, normalized to their peaks. Two AIFs have similar peak shapes but start to differ at the elimination phase as Gd-DOTA and ^18^F-Choline have different tissue uptake rates.

**Figure 1:**
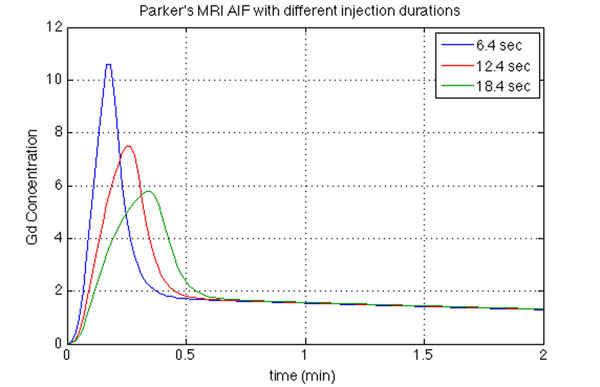
Simulated MRI-AIFs using Parker’s population-based input function refitted with the developed function. AIF shapes with different injection durations, Δτ is shown.

**Figure 2:**
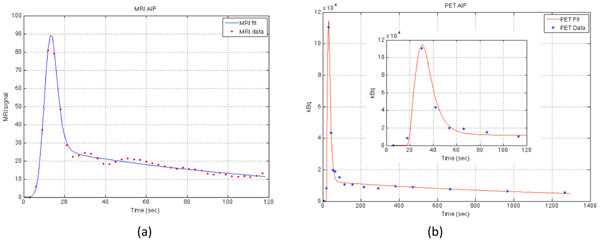
The double Butterworth convolution function used to fit (a) DSC-MRI data and (b) 18F-Choline PET data together with a plot where the timescale of PET-AIF was limited to MRI-AIF’s to show different bolus widths.

**Figure 3:**
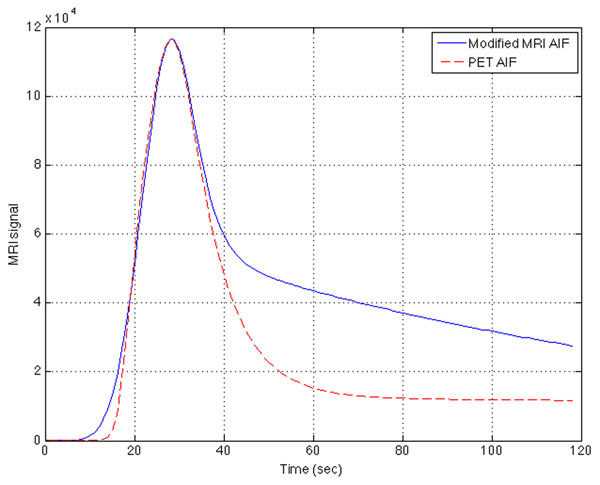
The MRI-AIF with modified τ1 and τ2 values plotted together with the PET-AIF. The MRI-AIF peak is scaled to PET-AIF’s peak.

This enables conversion of the early part of the AIFs from one modality to another even if different injection protocols are used.

## References

[1] Poulin E, Lebel R, Croteau E, Blanchette M, Tremblay L, Lecomte R, Bentourkia M, Lepage M (2013). Conversion of arterial input functions for dual pharmacokinetic modeling using Gd-DTPA/MRI and 18F-FDG/PET. Magn. Reson. Med.

[2] Holt A, Pasca E, Heijmink SW, Teertstra J, Muller SH, van der Heide UA (2012). The impact of overall injection time on the arterial input function and pharmaco-kinetic analysis using the Tofts model in DCE-MRI for prostate cancer patients. Proc. Intl. Soc. Mag. Reson. Med.

[3] **Perfusion Mismatch Analyzer, version 3.4.0.6 [January 20, 2014]** [http://asist.umin.jp/data-e.shtml] *ASIST-Japan Web site* 2006. updated June 2011

[4] Erlandsson K, Buvat I, Pretorius PH, Thomas B, Hutton BF (2012). A review of partial volume correction techniques for emission tomography and their applications in neurology, cardiology and oncology. Phys. Med. Biol.

[5] Parker GJM, Roberts C, Macdonald A, Buonaccorsi GA, Cheung S, Buckley DL, Jackson A, Watson Y, Davies K, G C, Jayson GC (2006). Experimentally-derived functional form for a population-averaged high-temporal-resolution arterial input function for dynamic contrast-enhanced MRI. Magn. Reson. Med.

